# An artificial intelligence study: automatic description of anatomic landmarks on panoramic radiographs in the pediatric population

**DOI:** 10.1186/s12903-023-03532-8

**Published:** 2023-10-17

**Authors:** İrem Bağ, Elif Bilgir, İbrahim Şevki Bayrakdar, Oğuzhan Baydar, Fatih Mehmet Atak, Özer Çelik, Kaan Orhan

**Affiliations:** 1 Department of Pediatric Dentistry, Faculty of Dentistry, Eskisehir Osmangazi University, Eskişehir, Turkey; 2 Department of Oral and Maxillofacial Radiology, Faculty of Dentistry, Eskisehir Osmangazi University, Eskişehir, Turkey; 3 Dentomaxillofacial Radiology Specialist, Faculty of Dentistry, Ege University, İzmir, Turkey; 4 Department of Computer Engineering, The Faculty of Engineering, Boğaziçi University, İstanbul, Turkey; 5 Department of Mathematics-Computer, Eskisehir Osmangazi University Faculty of Science, Eskisehir, Turkey; 6 Department of Oral and Maxillofacial Radiology, Faculty of Dentistry, Ankara University, Ankara, Turkey

**Keywords:** Anatomic landmarks, Artificial intelligence, Deep learning, Panoramic radiography, Pediatric dentistry

## Abstract

**Background:**

Panoramic radiographs, in which anatomic landmarks can be observed, are used to detect cases closely related to pediatric dentistry. The purpose of the study is to investigate the success and reliability of the detection of maxillary and mandibular anatomic structures observed on panoramic radiographs in children using artificial intelligence.

**Methods:**

A total of 981 mixed images of pediatric patients for 9 different pediatric anatomic landmarks including maxillary sinus, orbita, mandibular canal, mental foramen, foramen mandible, incisura mandible, articular eminence, condylar and coronoid processes were labelled, the training was carried out using 2D convolutional neural networks (CNN) architectures, by giving 500 training epochs and Pytorch-implemented YOLO-v5 models were produced. The success rate of the AI model prediction was tested on a 10% test data set.

**Results:**

A total of 14,804 labels including maxillary sinus (1922), orbita (1944), mandibular canal (1879), mental foramen (884), foramen mandible (1885), incisura mandible (1922), articular eminence (1645), condylar (1733) and coronoid (990) processes were made. The most successful F1 Scores were obtained from orbita (1), incisura mandible (0.99), maxillary sinus (0.98), and mandibular canal (0.97). The best sensitivity values were obtained from orbita, maxillary sinus, mandibular canal, incisura mandible, and condylar process. The worst sensitivity values were obtained from mental foramen (0.92) and articular eminence (0.92).

**Conclusions:**

The regular and standardized labelling, the relatively larger areas, and the success of the YOLO-v5 algorithm contributed to obtaining these successful results. Automatic segmentation of these structures will save time for physicians in clinical diagnosis and will increase the visibility of pathologies related to structures and the awareness of physicians.

## Background

Panoramic radiography is used for primary evaluations of the oral cavity for diagnosis of pathologies and treatment plans in dentistry [1]. It is possible to obtain information about the teeth, jawbones, sinuses, temporomandibular joints, and other hard tissues of the head and neck with panoramic radiography [2].

Panoramic radiographs, in which maxillary and mandibular anatomic structures can be observed, are used for the initial detection of cases closely related to the pediatric dentistry approach. Detection of dental anomalies and pathologies such as mesiodens, supernumerary teeth, odontoma, cysts, and impacted teeth on panoramic radiographs and determining their relationship with anatomic structures constitute a part of the first examination [3–6]. In pediatric patients for whom pain management is of great importance, injection of the local anesthetic solution near the mandibular foramen is a difficult anesthetic method to achieve, considering that the mandibular foramen position changes throughout the growth period [7]. Therefore, for a successful inferior alveolar nerve block, it is important to determine the position of the mandibular foramen correctly. In addition, the localization of the mandibular and mental foramen in children is an important factor to avoid damage to the neurovascular bundle in the management of complicated cases such as mandibular fractures in children [8, 9]. Mandible fractures constitute the majority (20–50%) of pediatric facial fractures, and condyle fractures are most common in the mandible. The risk of fracture is high, especially during the school-starting period. It is important to perform a panoramic X-ray in the first examination of these patients [10].

Artificial intelligence (AI) has been used for enhancing the analysis of dental radiology images. In the realm of two-dimensional (2D) radiographic images, digital radiographs are composed of myriad pixels, characterized by varying degrees of brightness and radiopacity. Artificial intelligence programs “learn” to analyze digital images by using these characteristics [11]. Deep learning is a machine learning method that excels in analyzing text and images [12]. Deep learning technique automatically learns the dataset using convolutional neural networks (CNN) which are a deep learning architecture, and creates a learning model after the learning process [13, 14]. There are many studies in the literature combining deep learning with dental radiography. Artificial intelligence technologies were used for the evaluation of pathological radiolucent lesions, diagnosis of osteoporosis, maxillary sinusitis, anatomic landmarks detection in adults and atherosclerotic plaques in carotid arteries on panoramic radiographs [15–19].

In some cases, when detailed examination of anatomic structures is required, it is possible to evaluate with three-dimensional imaging method, however, on clinical grounds, panoramic images are the primary available images for pediatric dentistry. Overlapping hard and soft anatomic landmarks (e.g. tongue, airway, nasal cavity flor, palate floor) on panoramic radiographs can make a difficult diagnosis [20]. Several factors contribute to the reliability of landmark identification in children: the definition of the landmark, the density and sharpness of images, the anatomic complexity of tissues, and the experience of the observers, especially for pediatric dentistry [2, 21].

Considering the advantages of showing the teeth and adjacent anatomic structures in a comprehensive way, being a fast and useful technique, easier application than intraoral techniques, and obtaining images with low radiation dose, AI-supported detection of pediatric anatomic landmarks in panoramic radiographs may prefer in the first examination in children. Artificial intelligence applications can serve as their guide to making better diagnoses and treatment plans with a low margin of error. It is thought that it will bring practicality to dentists [19]. To the best of our knowledge, although there have been a few studies on the mandibular canal and maxillary sinus, no comprehensive study has evaluated pediatric anatomic landmarks on panoramic radiographs using a deep-learning model. Therefore, the aim of the study was to investigate the success and reliability of the detection of anatomic landmarks observed on panoramic radiographs in children using artificial intelligence.

## Methods

### Data sources

In this study, in the pediatric patient population in the CranioCatch (Eskisehir, Turkey) database; A total of 981 mixed images for 9 different pediatric anatomic landmarks including maxillary sinus, orbita, mandibular canal, mental foramen, foramen mandible, incisura mandible, articular eminence, condylar and coronoid processes were labelled and were produced Pytorch-implemented YOLO-v5 models. This study was conducted in accordance with the code of ethics of the World Medical Association (Declaration of Helsinki). The approval of this study was granted by Eskisehir Osmangazi University Non-interventional Clinical Research Ethics Board, Eskişehir, Turkey which also approved informed consent (decision no. 04.10.2022/22). An informed consent was obtained from all subjects and/or their legal guardian(s).

Images of individuals in the pediatric population were included. Radiographic images obtained by incorrect positioning of the patient or containing motion artifacts were excluded from the study. Age, gender, and ethnicity differences were not observed, and the data was anonymized prior to uploading into the labeling system.

### Labelling and training of data

Labelling is the process of identifying areas in an image and determining which region the object belongs to. Labelling; was carried out using web-based CranioCatch (Eskişehir, Turkey) software by oral and maxillofacial radiologist and a pediatric dentist with at least 10 years of experience. For labelling, anatomic regions were selected in the panoramic radiograph. The outer boundaries of these anatomical regions were established through polygonal segmentation and stored in JSON (JavaScript Object Notation) format, following a precise delineation process. Segmentation model 981 anonymized, mixed-sized panoramic radiography images were resized to 640 × 320 diameter. In this study, the training was executed employing the PyTorch library in Python. To ensure the integrity of the evaluation process, the dataset was partitioned into three distinct subsets: 80% designated for training purposes, 10% for validation to fine-tune parameters, and another 10% for final model testing.

- Training group: 80% of the images (data set for training the model).
- Validation group: 10% of the images (Denotes the set of instances that remain dissociated from the model’s training process and must remain concealed from the model’s purview throughout this phase. This subset is employed to assess the model, determining whether to halt the training or modify training parameters.)
- Test group: 10% of the images (This subgroup was used to assess how well the trained model worked, using both the training and validation data.)

The training and validation datasets were important for figuring out the best settings for the AI algorithm. We then checked the success of the model performed using the test dataset.

### Deep-learning algorithm

The images, after undergoing classification and labeling, were resized to dimensions of 640 × 320 pixels as part of the training process. In this study, the training phase was conducted using the PyTorch library within the Python programming language, employing 2D CNN architectures, and encompassing a span of 500 training epochs. For the segmentation task, the YOLO-v5 (Ultralytics, San Francisco, California, USA) was used. YOLO-v5, a member of the computer vision model lineage, offers a quartet of progressive versions, namely: small (s), medium (m), large (l), and extra-large (x). We used the YOLO-v5x model here. The architecture of YOLO-v5x comprises three fundamental constituents: Backbone, Neck, and Head. Notably, CSPDarknet53 serves as the foundational backbone. The Neck section integrates the Path Aggregation Network (PANet) and Spatial Pyramid Pooling (SPP) techniques, facilitating the fusion of extracted features. Subsequently, the YOLO Layer culminates in segmentation outcomes, encompassing class categorizations, confidence scores, and spatial coordinates. Upon the development of YOLO models, the PyTorch model’s outputs were subjected to assessment on a reserved 10% subset of the dataset, yielding insightful accuracy metrics.

The model’s effectiveness underwent assessment via a confusion matrix, a graphical depiction of the juxtaposition between predicted and actual situations. The model’s efficacy was gauged through the computation of performance metrics, derived from the interplay of True Positive (TP: accurate diagnoses correctly detected and segmented), False Positive (FP: diagnoses mistakenly identified and imprecisely segmented), and False Negative (FN: diagnoses incorrectly detected and segmented) evaluations. This evaluative framework encompassed metrics such as sensitivity, precision, and the F1 score—a harmonic medium of these indices.

## Results

14,804 labels a total of 981 mixed images were made for 9 different pediatric anatomic landmarks including maxillary sinus (1922), orbita (1944), mandibular canal (1879), mental foramen (884), foramen mandible (1885), incisura mandible (1922), articular eminence (1645), condylar (1733) and coronoid (990) processes. F1 score and sensitivity values for these anatomic regions were found as 0.98–0.99, 1–1, 0.97–0.99, 0.88–0.92, 0.95–0.95, 0.99–0.99, 0.92–0.92, 0.94–0.99, 0.86–0.97 respectively (Table 1).

**Table 1:** The sensitivity, precision, and F1 Score value of AI model estimation performance measures

	Measurement Value		
	F1 Score	Precision	Sensitivity
**Maxillary sinus**	0.98	0.97	0.99
**Orbita**	1	1	1
**Mandibular canal**	0.97	0.94	0.99
**Mental foramen**	0.88	0.84	0.92
**Foramen mandible**	0.95	0.96	0.95
**Incisura mandible**	0.99	0.98	0.99
**Articuler eminence**	0.92	0.93	0.92
**Condylar processes**	0.94	0.89	0.99
**Coronoid processes**	0.86	0.76	0.97

**Fig. 1:**
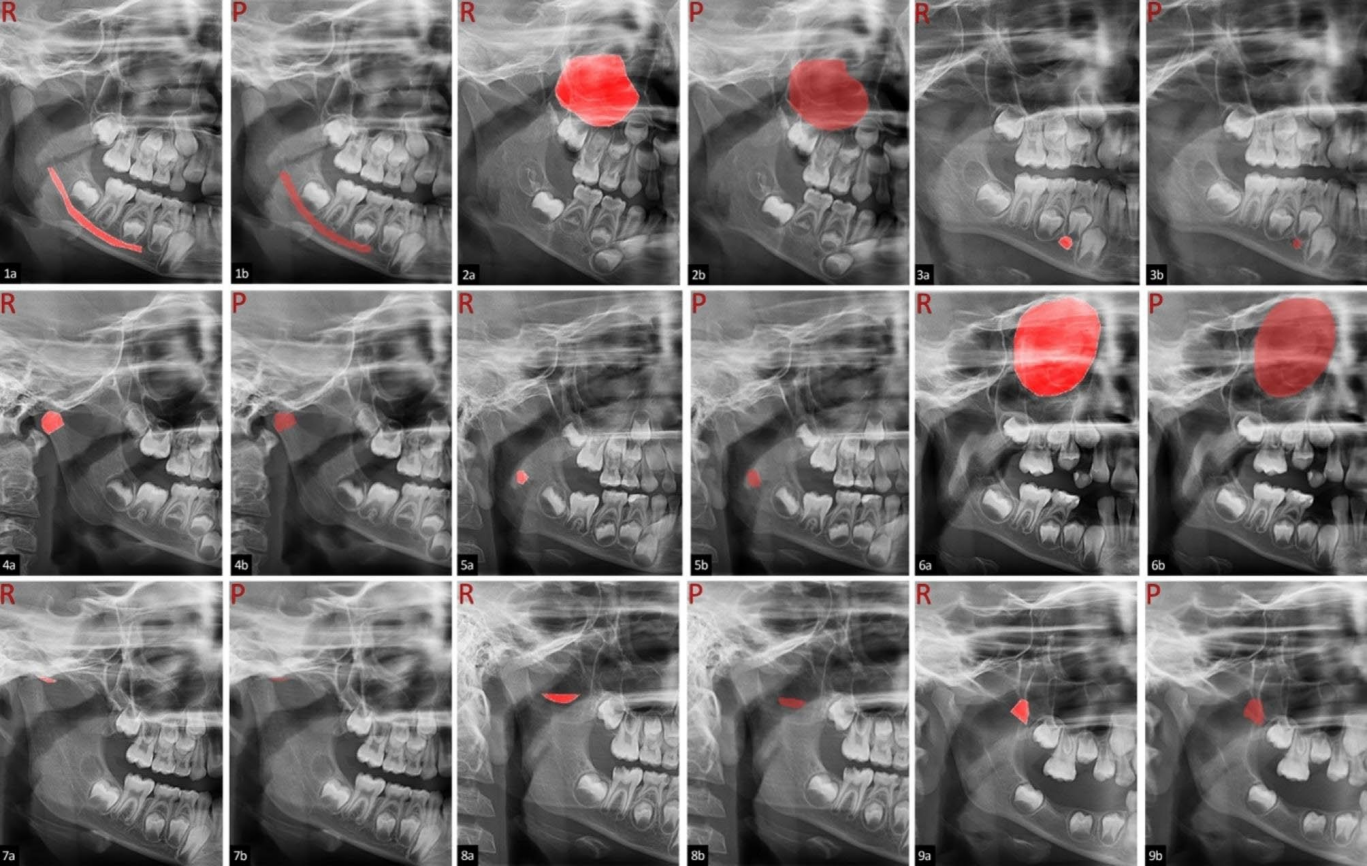
True positive real (R) and predictive (P) images for pediatric anatomic landmarks (mandibular canal 1a-1b, maxillary sinus 2a-2b, mental foramen 3a-3b, condylar processes 4a-4b, foramen mandible 5a-5b, orbita 6a-6b, articular eminence 7a-7b, incisura mandible 8a-8b, coronoid process 9a-9b)

**Fig. 2:**
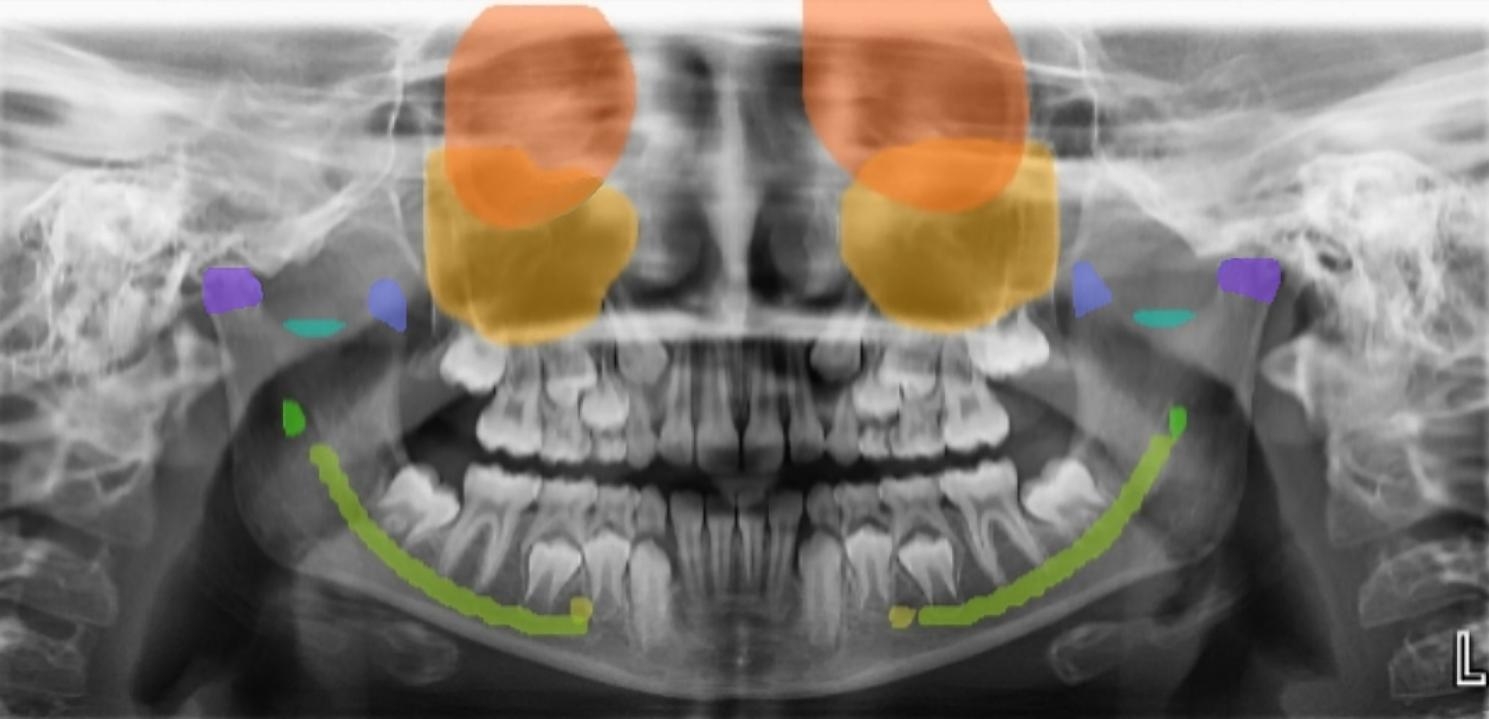
The estimation output of the AI model for all landmarks on a panoramic image

True positive real and predictive images for pediatric anatomic landmarks are shown in Fig. 1. The estimation output obtained as a result of the detection of all landmarks by the AI model on a panoramic image is shown in Fig. 2.

## Discussion

Panoramic radiography is the most commonly chosen imaging technique in which collaboration can be easily established in the pediatric patient group due to its simple and painless procedure. In this study, it aimed to evaluate the use of artificial intelligence technology to facilitate the detection of anatomic structures on panoramic radiographs in pediatric patients.

A systematic review reported that AI technology is widely used in different specialities of dentistry. AI technology has been used in oral and maxillofacial radiology, orthodontics, dentofacial orthopaedics, endodontics, periodontics, and oral and maxillofacial surgery [19]. CNNs have often been applied in orthodontics to help detect landmarks on lateral cephalometric radiographs [18, 22–24]. Dental diagnostic image analysis can also assist other medical professionals in diagnosing lesions and diseases [25–28]. To perform tooth numbering using deep learning algorithms on digital dental photographs, and to evaluate the success of these algorithms in determining the presence of frenulum, gingival hyperplasia and gingival inflammation. Yolov5 architecture were used in the creation of the models [29]. In the field of dentistry, the successful outcomes achieved in research utilizing artificial intelligence underscore its significant contributions, paving the way for transformative advancements in dental practice. These studies represent a crucial step towards accelerating the transformation of dentistry through the application of cutting-edge technology.

In addition to these advancements in dentistry, it’s worth noting that artificial intelligence (AI) technology has also become a new trend in forensic medicine, marking a watershed moment for the entire field of forensic science. In research aimed at facilitating interdisciplinary collaboration between forensic experts and deep learning engineers, a new workflow has been introduced for the 3D CNN analysis of full-head and neck cone-beam computed tomography (CBCT) scans. The application methods of 3D CNN for forensic investigations have yielded successful results in areas such as gender determination, estimation of biological age, annotation of 3D cephalometric landmark points, prediction of growth vectors, and estimation of facial soft tissue from the skull [30]. Age and gender assessment is used to identify victims, determine criminal responsibility, or identify individuals without legal documents [31, 32]. Studies using dental orthopantomogram examples, compared logistic regression linear models with neural networks for each legal age limit (14, 16, and 18 years) and showed that neural networks performed better [33, 34]. Similar studies in this field have favored neural networks for age prediction using dental orthopantomograms or landmarks in the mandible [35, 36]. It is certain that artificial intelligence will shape new developments and provide contributions in the field of forensic science.

Today, CBCT is a modern dental imaging system that provides rapid volumetric imaging with low radiation exposure. It has added a third dimension to the diagnostic process, surpassing manual and digital 2D analyses [37, 38]. Automated CBCT segmentation stands out as one of the current trends and innovations [39]. Undoubtedly, compared to the past when there were only 2D images such as panoramic or lateral X-rays, we have reached a higher level with CBCT and artificial intelligence. The benefits of using artificial intelligence for medical diagnosis in evaluating CBCT scans include improved accuracy, speed, and efficiency in diagnosis [40]. Recent studies evaluating the accuracy of AI-supported automatic detection of small edentulous areas in CBCT scans have shown that AI algorithms are fast and highly accurate in detecting teeth and small edentulous areas, tooth labelling is much faster, and segmentation is performed up to 900 times faster than when done by an experienced dental surgeon [41, 42].

There are some limitations to the processing of CBCT data by artificial intelligence [43], which can be divided into two main groups. Firstly, when considering the quality assessment of the literature, insufficient sample sizes and incomplete reporting are the main contributors to the high risk of bias. Secondly, AI techniques encompass highly heterogeneous applications in terms of dataset acquisition, analysis, and performance metrics, making comparisons challenging. Additionally, limitations such as AI algorithms affected by metallic artifacts [44, 45], variations in canal calcifications, errors due to examiner experience [46], and minimal cross-sectional areas [42, 45] are present in the processing of CBCT data by artificial intelligence.

Within the realm of dentistry, an investigation delved into the utilization of deep learning to segment the mandibular canal in panoramic radiography, yielding notably precise findings (with an accuracy of 0.847). This remarkable precision underscores the advantages of training on two-dimensional images, given that the canal prominently features in the 2D panoramic image, and the resolution of panoramas exceeds that of CBCT [47]. In a study aimed at improving the visualization of the Inferior Alveolar Canal (IAC), the edges of dental panoramic images were enhanced using a novel structural filter. Candidate regions were selected from the enhanced image by the proposed Multi Hidden Layer Extreme Learning Machine Artificial Neural Network (MELMANN) model. Experimental results indicate that this method effectively delineated the IAC [48].

In another study evaluating the success of different deep learning methods in detecting the mandibular canal with CBCT in adult patients, three-dimensional U-net was found to be the most successful. CBCT is an imaging modality that offers three-dimensional depth away from superpositions, therefore, many anatomical and pathological features can be defined more easily, and it can provide less complex sectional images for deep learning methods [49]. However, it is not an imaging method that can be used frequently in terms of radiation dose in the pediatric population. In the present study, models of the mandibular canal in panoramic radiographs were developed with a new-generation deep learning method, and success metrics were found to be high.

In dental panoramic X-ray images, it’s harder to see the mental foramen for patients under 20 years old, when compared to patients who are over 20 years old. Researchers have achieved a 95.4% mean dice similarity coefficient (DSC) for patients between 7 and 20 years of age working on 1000 dental pantomograph with the U-net model [50]. A study for segmentation of the mental foramen from 112 digital Panoramic x-ray images used a single full CNN based on U-net. Researchers reported the highest precision value 71.13% in this study [51]. In our data, it was difficult to distinguish the mental foramen, so we performed the training with the labels we made in about half of the total data set. The precision of the mental foramen model was lower than the other models, but we got a score of 0.84% here as well. This result is also promising for cases with difficult model detection. We think that both the number of mental foramen labels in panoramic radiography and the YOLO-v5, which we used in the development of the models, increased the success rate.

In a study, the researhers discovered that a deep learning system’s ability to diagnose maxillary sinusitis using panoramic X-rays was comparable to radiologists and even better than dental assistants [16]. Another research used deep learning to find and categorize issues in the maxillary sinus through panoramic X-rays. Like our study, they achieved a 100% success rate in detecting both normal and inflamed maxillary sinuses, mirroring our sensitivity results. The results showed that the maxillary sinus can be reliably detected in clinical practice with this AI model [14].

Particularly in pediatric patients, condylar fractures and displacement of the fractured condyle are frequently encountered as an indirect effect of a blow to the symphysis due to a face-down fall. This position change may be overlooked in trauma patients, and problems such as being late for treatment and stabilization cause displaced fragments to fuse in abnormal positions. In a study of panoramic radiographs of 100 condyles with and without mandibular condyle fractures from two hospitals to evaluate deep learning models, the system has been recommended for diagnosing condylar fractures as high classification performance was obtained, with operating characteristic curve values of > 0.85 and sensitivity 83–85% of data from both hospitals [52]. YOLOv5 were trained to automate the placement of the bounding boxes to detect fractures in the radiographic images. CNN-based models detected mandibular fractures with a performance surpassing that of experts [53]. The present study including 745 panoramic images, 0.99 sensitivity and 0.94 F1 score indicated that the YOLO-v5 model can be used successfully for mandibular automatic condyle segmentation.

As a limitation of the study, success may be low due to reasons such as the superposition of tooth germs in the mental foramen region, mixed dentition, and the presence of different densities in the relevant region. By increasing and diversifying the number of data, the success in detecting mental foramen should be increased with further studies.

## Conclusion

In this study, we developed YOLO-v5 models that will provide automatic detection of 9 important anatomic formations in approximately one thousand panoramic radiographs of pediatric patients. The success of correct detection of the models was found to be quite high when compared to the literature. Superposition of anatomic formations on panoramic radiographs may cause difficulties in dentomaxillofacial diagnosis. Knowing the anatomy helps to distinguish these structures from pathologies in the first place, then it is important in many ways such as clarifying the relationship of the related structures with the dental structures, creating awareness about the pathological conditions of these structures, monitoring the findings of the dental structures in these regions or the effects of the structures on the dental structures. The development of systems that automatically segment these structures in the graphs will play a role as clinical decision-support mechanisms, increase the awareness of physicians and save time at the same time.

## Data Availability

The datasets generated and/or analysed during the current study are not publicly available due [The data were obtained from anonymized radiographs by labelling them in a way that they cannot be accessed by reverse engineering. An informed consent form was obtained for the use of data for scientific purposes. We do not have patient consent to share raw data, in addition, the data is anonymized to not contain personal information.] but are available from the corresponding author on reasonable request.

## List of abbreviations

CNN: Convolutional neural networks
AI: Artificial intelligence
2D: Two-dimensional
JSON: JavaScript Object Notation
PANet: Path Aggregation Network
SPP: Spatial Pyramid Pooling
TP: True positive
FP: False positive
FN: False negative
CBCT: Cone beam computed tomography
DSC: Dice similarity coefficient
IAC: Inferior Alveolar Canal
